# Protons Potentiate GluN1/GluN3A Currents by Attenuating Their Desensitisation

**DOI:** 10.1038/srep23344

**Published:** 2016-03-22

**Authors:** Kirstie A. Cummings, Gabriela K. Popescu

**Affiliations:** 1 Department of Biochemistry, University at Buffalo, SUNY, Buffalo, NY 14214, USA

## Abstract

N-methyl-D-aspartate (NMDA) receptors are glutamate- and glycine-gated channels composed of two GluN1 and two GluN2 or/and GluN3 subunits. GluN3A expression is developmentally regulated, and changes in this normal pattern of expression, which occur in several brain disorders, alter synaptic maturation and function by unknown mechanisms. Uniquely within the NMDA receptor family, GluN1/GluN3 receptors produce glycine-gated deeply desensitising currents that are insensitive to glutamate and NMDA; these currents remain poorly characterised and their cellular functions are unknown. Here, we show that extracellular acidification strongly potentiated glycine-gated currents from recombinant GluN1/GluN3A receptors, with half-maximal effect in the physiologic pH range. This was largely due to slower current desensitisation and faster current recovery from desensitisation, and was mediated by residues facing the heterodimer interface of the ligand-binding domain. Consistent with the observed changes in desensitisation kinetics, acidic shifts increased the GluN1/GluN3A equilibrium current and depolarized the membrane in a glycine concentration-dependent manner. These results reveal novel modulatory mechanisms for GluN1/GluN3A receptors that further differentiate them from the canonical glutamatergic GluN1/GluN2 receptors and provide a new and potent pharmacologic tool to assist the detection, identification, and the further study of GluN1/GluN3A currents in native preparations.

NMDA receptors are members of the ionotropic glutamate receptor (iGluR) family. They form dimers of heterodimers, which consist of an obligatory GluN1 subunit, and a GluN2 or a GluN3 subunit. Wide molecular diversity results from alternative splicing of the obligatory GluN1 subunit transcript (1a–4b)^1^,^2^ and from differential expression of six genes: GluN2 (A–D) and GluN3 (A, B)^3^,^4^,^5^,^6^. All iGluR subunits have similar topology and consist of a modulatory N-terminal domain (NTD) and a ligand/agonist-binding domain (LBD) located externally; a pore-forming membrane-spanning domain; and a C-terminal domain (CTD) located internally. In most neurons in the central nervous system, two glycine-binding GluN1 and two glutamate-binding GluN2 subunits form glycine- and glutamate-gated excitatory channels that have characteristically slow kinetics and high calcium permeability; these are essential for normal synaptic development and plasticity and are strongly inhibited by physiologic transients of extracellular H^+^ and Zn^2+^ (ref. 7). When two glycine-binding GluN1 subunits assemble with two glycine-binding GluN3A subunits, the resulting excitatory channels are gated by glycine alone, are insensitive to glutamate or NMDA, and are potentiated by extracellular Zn^2+^ (ref. 8,9). Dysregulated expression of GluN3A has been linked to cognitive and neurodegenerative conditions, including schizophrenia and ischemia^10^,^11^,^12^,^13^, but the operation and biological functions of GluN3A-containing receptors remain elusive.

In brain interstitia, pH fluctuates during normal synaptic transmission and is altered in several pathologic conditions. During synaptic activity, vesicular co-release of glutamate and protons can briefly reduce the local pH by 0.2–0.25 units^14^; mild but persistent global acidification (~0.2 units) occurs in schizophrenia^15^,^16^,^17^; and focused but more dramatic shifts (~0.8 units) occur after ischemic events^18^. Such pH fluctuations influence the activity of several neuronal receptors, including the canonical NMDA receptors, whose activity is half-maximal at physiologic pH^19^.

Although all functional iGluRs have similar modular architectures, functional features of structural modules are not strictly conserved across members. For example, in all iGluRs, agonists bind in the cleft formed by the two hinged lobes that comprise the LBD of each subunit, and the degree of cleft closure correlates with agonist efficacy^20^,^21^,^22^. In contrast, the surface that joins two LBDs into a dimer is the locus of desensitisation only in non-NMDA-type iGluRs^23^,^24^,^25^; additionally, synaptic modulators, including H^+^ and Zn^2+^, generally act within the LBD of non-NMDA receptors but act within the NTD of NMDA receptors^26^,^27^,^28^.

Here, we report that small acidic deviations from physiologic pH strongly increased glycinergic GluN1/GluN3A currents, mainly by slowing their desensitisation and accelerating their resensitisation. Results from our structure-based mutagenesis indicated that this effect occurred mainly by an allosteric mechanism mediated by protonatable residues facing the LBD dimer interface, which likely stabilize the heterodimer. Last, we show that extracellular acidification increased tonic GluN1/GluN3A currents and depolarized the cellular membrane potential in a glycine concentration-dependent manner. These results indicate functional conservation of the dimer interface of GluN1/GluN3A receptors with that of non-NMDA iGluRs, and reveal novel modulatory mechanisms in GluN1/GluN3A receptors that may be important for their cellular functions *in vivo*. Importantly, our results provide a new and needed pharmacologic tool to identify glycinergic GluN1/GluN3A currents in native cells and tissues.

## Results

### Protons potentiate GluN1/GluN3A responses strongly and selectively

**Figure 1:**
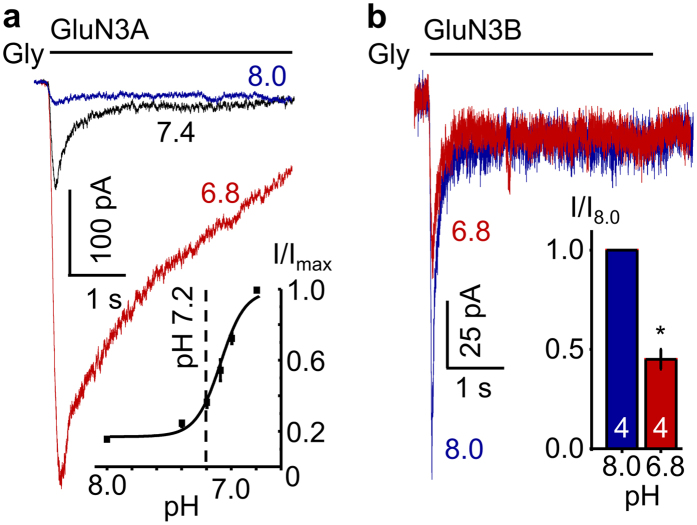
Protons potentiate GluN3A- but not GluN3B-containing glycinergic NMDA receptor currents. Representative glycine-elicited whole-cell currents (0.5 mM, −70 mV) at the indicated pH values from HEK293 cells expressing: (**a**) GluN1-1a/GluN3A receptors; *inset* illustrates the proton dose dependency of the change in peak current amplitude relative to I_max_, which occurred at pH 6.8 (n = 24). Dotted line indicates physiological brain pH (7.2); or (**b**) GluN1-1a/GluN3B receptors; *inset* summarizes the results; values ae means ± SEM; sample size is in bars; *P < 0.05.

We expressed recombinant GluN1-1a/GluN3A receptors in HEK293 cells, and elicited whole-cell currents with glycine (0.5 mM, 5 s) (EC_50_ ~50 μM)^29^ at several external pH levels. In physiologic pH, glycine produced deeply desensitising currents, as reported previously^29^,^30^,^31^. However, we found that the peak current amplitude (I_pk_) increased steeply with extracellular acidification. The maximum peak current (I_max_) occurred at pH 6.8 and was ~3-fold larger than the peak current recorded at pH 7.2 and ~6-fold larger than that at pH 8.0, with a half-maximal effect observed at pH 7.10 ± 0.03 (Fig. 1a, n = 24). This behaviour is in sharp contrast with the well-established inhibition of GluN1-1a/GluN2 currents by external acidification (IC_50_ = pH 7.2)^19^. This unexpected result suggests that fluctuations in external pH may have substantial and opposing effects on the excitatory currents produced by GluN1/GluN2 and GluN1/GluN3 receptors.

To investigate whether protons potentiate GluN1/GluN3A currents specifically, or whether this modulation extends to GluN1/GluN3B currents as well, we next examined the pH-dependence of glycinergic currents recorded from HEK293 cells expressing GluN1-1a/GluN3B receptors. Using the same experimental conditions as above, we found that, as reported previously for receptors expressed in oocytes^28^,^32^, these currents were inhibited by acidification, such that the peak current recorded at pH 6.8 was ~2-fold smaller relative to that recorded at pH 8.0 (n = 4, P = 0.001, paired Student’s t-test) (Fig. 1b). Together these results show that within the NMDA receptor family, glycinergic GluN1/GluN3A currents are robustly and uniquely potentiated by external acidification within the physiologic range of pH fluctuations.

### Protons control the kinetics of GluN1/GluN3A current desensitisation and recovery

**Figure 2:**
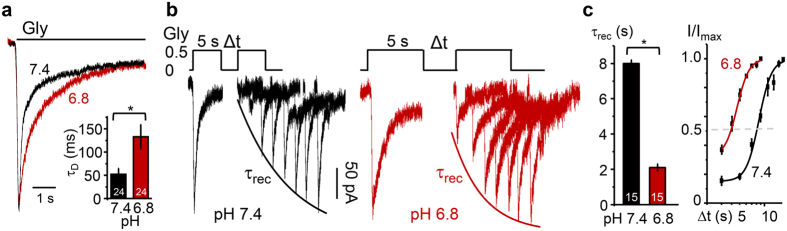
Protons alter GluN1/GluN3A desensitisation kinetics. (**a**) Glycine-elicited currents recorded at pH 7.4 (black) and pH 6.8 (red) superimposed and normalized to peak; bar graph summarizes changes in the weighted desensitisation time course (τ_D_). (**b**) Glycine double-pulse current traces elicited at pH 7.4 and pH 6.8; Δt was 3 s with 2 s increments for pH 7.4, and 3 s with 1 s increments for pH 6.8; curves represent exponential fits to average I_pk_ values. (**c**) Summary data for time constant of recovery from desensitisation (τ_rec_) (left) and time-dependence of peak current (I_pk_) recovery from desensitisation (right). Values are means ± SEM, sample size is in bars; *P < 0.05.

To investigate potential mechanisms by which protons increased GluN1/GluN3A current amplitudes, we examined the response time course during sustained exposure to glycine (0.5 mM, 5 s) in several external pH levels and measured desensitisation kinetics as the time between peak (I_pk_) and steady-state (I_ss_) current amplitudes, which followed a biphasic exponential function. We determined that the weighted time constant (τ_D_) increased ~3-fold, from 52 ± 13 ms at pH 7.4 to 133 ± 26 ms at pH 6.8 (n = 24, P = 0.008, paired Student’s t-test) (Fig. 2a and Table 1). Therefore, protonation slowed substantially the macroscopic current desensitisation. Next, we asked whether, in addition to slowing entry into desensitisation, acidification also affected the time with which the response recovered from desensitisation.

We examined resensitisation kinetics with a two-pulse protocol, consisting of an initial desensitising pulse followed at increasing intervals of time by a test pulse^33^ as follows. Receptors were perfused extensively (>25 s) with glycine-free external solution to synchronise them into resting agonist-free conformations; they were next stimulated with a desensitising 5-s pulse of glycine (0.5 mM), which produced currents that peaked to a maximum amplitude (I_max_) and declined to an equilibrium level (I_ss_). Next, we applied glycine-free solutions for increasingly longer periods (Δt, 3 s–21 s), which allowed peak currents to recover to various degrees; finally, a second pulse of glycine (0.5 mM) was applied to reveal the extent of recovery of the peak current (I) (Fig. 2b). We found that external pH markedly influenced resensitisation kinetics. The recovery time constant was τ_rec_, 8.0 ± 0.2 s at pH 7.4 and 2.1 ± 0.2 s at pH 6.8 (n = 15, P = 0.0001, paired Student’s t-test); and the recovery half time was t_1/2_, 8.4 ± 0.3 s at pH 7.4 and 4.6 ± 0.2 s at pH 6.8 (n = 15, P = 0.001, paired Student’s t-test) (Fig. 2c and Table 1). Together these kinetic measurements show that acidification caused currents to desensitise slower and to recover faster, such that shifting the external pH from pH 7.4 to pH 6.8 increased the acute response (I_pk_) ~3-fold and the equilibrium or tonic responses (I_ss_) ~2-fold. Next, we sought to elucidate the molecular determinants responsible for this effect.

### Proton-dependent potentiation is independent of NTD and CTD residues

**Figure 3:**
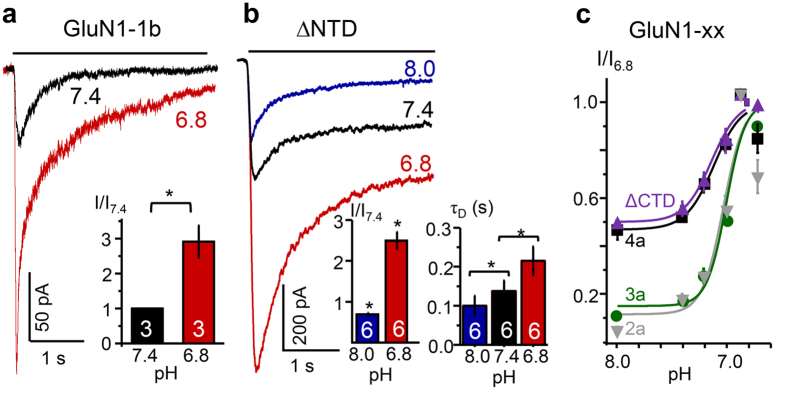
Proton potentiation is independent of residues in the NTD and CTD layers. Glycine-elicited whole-cell currents (0.5 mM, −70 mV) recorded at the indicated pH values from HEK293 cells expressing: (**a**) GluN1-1b/GluN3A receptors or (**b**) receptors lacking the entire NTD layer (ΔNTD). (**c**) pH-dependence of I_pk_ relative to pH 6.8 for receptors with the indicated GluN1 CTD sequences (▿, GluN1-2a (2a); ○, GluN1-3a (3a); □ GluN1-4a (4a); Δ, GluN1^838stop^ (ΔCTD)). Values are means ± SEM; sample size is in bars; *P < 0.05 relative to pH 7.4.

Uniquely among iGluRs, NMDA receptors have several physiologically important allosteric sites located in the NTD. In particular, the proton sensitivity of GluN1/GluN2 receptors is strongly attenuated when a stretch of positively charged residues encoded by the alternatively spliced exon 5 is included in the NTD region of GluN1 transcript^28^. Relative to exon 5-lacking GluN1-1a/GluN2A receptors, exon 5-inclusive GluN1-1b/GluN2A receptors are 4-fold less sensitive to proton inhibition (IC_50_, 270 *vs.* 60 nM)^28^. To investigate whether inclusion of exon 5 influences GluN1/GluN3A receptor sensitivity to extracellular pH fluctuations, we recorded glycine-elicited currents from GluN1-1b/GluN3A receptors at pH 7.4 and pH 6.8. We found that despite expressing the charged sequence encoded by exon 5, GluN1-1b/GluN3A receptors remained highly sensitive to proton potentiation, producing peak currents that were ~3-fold larger at pH 6.8 relative to pH 7.4 (n = 3, P = 0.001, paired Student’s t-test) (Fig. 3a). Moreover, when we examined receptors that lacked the entire NTD layer (GluN1-4a^ΔN^/GluN3A^ΔN^; Fig. 3b) we found that relative to pH 7.4, they produced currents that were 2.6 ± 0.2-fold larger (n = 6, P = 0.001, paired Student’s t-test). We noticed that removing the NTD layer slowed current desensitisation substantially; however, protons remained effective and further slowed these kinetics, increasing τ_D_ from 0.10 ± 0.03 s in pH 8 to 0.22 ± 0.04 s in pH 6.8 (n = 6, P = 0.002, paired Student’s t-test) (Fig. 3b). These results indicate that NTD residues modulate the gating kinetics of GluN1/GluN3A receptors however, they are not necessary for the potentiating effect of protons.

In addition to the NTD variants tested above (denoted a and b), differential splicing of the GluN1 transcript also produces NMDA receptors that differ in their CTD sequence (denoted 1 – 4)^1^,^2^. To determine whether these naturally occurring variants produce currents that are also sensitive to proton potentiation, we co-expressed these in turn with GluN3A in HEK293 cells, and recorded glycine-elicited (0.5 mM, 5 s) whole-cell currents at several pH levels. We found that regardless of CTD sequence, GluN1/GluN3A currents were potentiated by acidic pH with similar EC_50_ values: GluN1-2a, 7.01 ± 0.04 (n = 10); GluN1-3a, 7.03 ± 0.05 (n = 8), and GluN1-4a, 7.01 ± 0.04 (n = 5) (Fig. 3c). Together with the NTD variants examined above, these results show that all naturally occurring GluN1/GluN3A receptor variants have similar pH sensitivities and, except for receptors containing GluN1-4a, whose currents were potentiated only ~2-fold, all others were potentiated ~3- to 4-fold by pH shifts in the 7.4 to 6.8 range.

Further, we found that receptors that lacked the entire GluN1 C-terminus^34^, GluN1^838stop^/GluN3A, responded to changes in external pH in a manner similar to receptors containing the GluN1-4a subunit (Fig. 3c, ΔCTD). Therefore, we conclude that residues in the GluN1 CTD can modulate the extent of proton-dependent potentiation but are not required for this effect. We could not measure currents from receptors lacking the GluN3A CTD, most likely due to deficits in receptor trafficking to the plasma membrane^35^. Therefore, we could not ascertain whether this region modulates the receptor’s potentiation by external pH. Next, we turned to the LBD layer as a possible host for the proton-binding site(s) of GluN1/GluN3A receptors.

### Protons potentiate GluN1/GluN3A currents through a novel allosteric site

**Figure 4:**
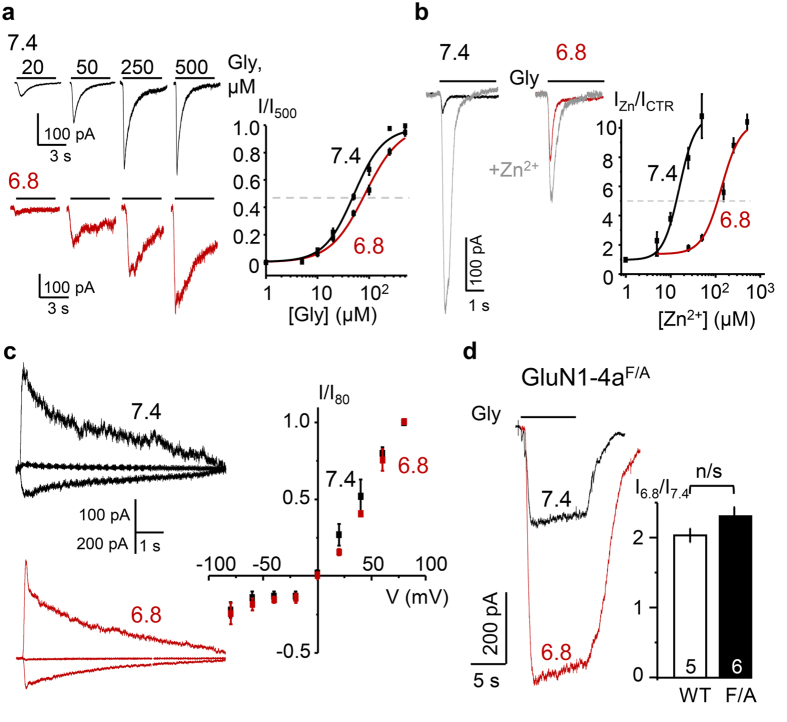
Protons alter sensitivities to LBD ligands and act through a novel site. (**a**) Glycine-dose dependency of I_pk_, at two pH values. (**b**) Glycine-elicited currents recorded in the continuous presence (50 μM, grey) or absence (0 μM, black or red) of external Zn^2+^ at two pH values, and zinc-dose dependency of I_pk_ change relative to 0 Zn^2+^ (I_CTR_), at two pH values. (**c**) Glycine-elicited currents recorded with 1.8 mM external Ca^2+^ at several holding potentials, and the voltage dependence of I_pk_ relative to +80 mV (I_80_) at two pH values. (**d**) Glycine-elicited currents from cells expressing GluN1-4a^F484A^ and GluN3A (GluN1-4a^F/A^) at two pH values and summary of changes in I_pk_. Values are means ± SEM; sample size is in bars; n/s, P > 0.05.

Within the iGluR family, many members have allosteric modulatory sites in the LBD layer and several are well characterised structurally and functionally^36^. Among these, Zn^2+^-binding to the LBD of homomeric GluK3 kainate receptors potentiates currents and modulates agonist sensitivity^26^. Zn^2+^ also potentiates glycine-elicited GluN1/GluN3A currents, and the Zn^2+^-binding site most likely resides in the LBD^9^. Therefore, as a first pass inquiry into the role of GluN1/GluN3A LBD residues in pH modulation, we asked whether changes in external pH affect the receptors’ sensitivity to the LBD ligands glycine and Zn^2+^. We found that external acidification decreased sensitivity (increased EC_50_) for glycine 2-fold, from 48 ± 6 μM at pH 7.4 (n = 4) to 83 ± 3 μM at pH 6.8 (n = 7) (Fig. 4a), and for Zn^2+^ 8-fold, from 16 ± 2 μM at pH 7.4 (n = 6) to 133 ± 12 μM at pH 6.8 (n = 16) (Fig. 4b). Notably, at pH 6.8, where proton potentiation was maximal, Zn^2+^ remained effective and further increased peak currents 2-fold. These results are consistent with a role of LBD residues in mediating proton potentiation of GluN1/GluN3A receptors, and suggest that separate residues mediate the H^+^ and Zn^2+^ effects.

To investigate further whether H^+^ and Zn^2+^ act through the same or overlapping residues of GluN1/GluN3A receptors, we examined how external pH affected the following two Zn^2+^-dependent functional phenotypes: the Zn^2+^-dependent relief from Ca^2+^-dependent outward rectification of GluN1/GluN3A current-voltage relationship (I/V)^37^ and the resistance of GluN1^F484A^/GluN3A currents to Zn^2+^-dependent potentiation^9^. First, we examined H^+^-dependent effects on Ca^2+^-dependent current/voltage relationship by measuring the voltage dependency of GluN1/GluN3A currents in the presence of external Ca^2+^ (1.8 mM) at pH 7.4 and pH 6.8. We found that, in contrast to Zn^2+^, which restores a linear I/V relationship^37^, increasing extracellular proton concentration did not alter the I/V profile (n = 4) (Fig. 4c). This result indicates that unlike Zn^2+^, protons had no effect on Ca^2+^-dependent block of GluN1/GluN3A inward currents, thus suggesting that Zn^2+^ and H^+^ modulate GluN1/GluN3A currents by separate mechanisms.

Second, we tested whether pH modulated currents recorded from a Zn^2+^-insensitive mutant, GluN1^F484A^/GluN3A. A single residue substitution in the glycine-binding site of the GluN1 subunit (GluN1^F484A^) causes a marked (~6,000-fold) reduction in the glycine sensitivity of glutamatergic GluN1/GluN2A currents^9^. The same mutation causes a large increase in GluN1/GluN3A glycinergic currents, and renders receptors insensitive to further potentiation by Zn^2+^ (ref. 9). We reasoned that, if Zn^2+^ and H^+^ act on the same site(s), we should observe a similar insensitivity to H^+^-dependent potentiation of currents recorded from this mutant. To investigate, we recorded whole-cell currents from GluN1^F484A^/GluN3A receptors expressed in HEK293 cells at pH 7.4 and pH 6.8 and found that, as reported previously in physiologic pH from oocytes^9^, these currents were much larger than those recorded from wild-type receptors. In addition, we observed that these currents desensitised to a much lower extent (Fig. 4d), indicating that the mutation affected desensitisation kinetics. However, despite these marked changes in gating, extracellular acidic pH shifts further increased currents recorded from GluN1^F484A^/GluN3A: I_6.8_/I_7.4_, 2.2 ± 0.3 (n = 6, P = 0.004, paired Student’s t-test), and the magnitude of the effect was similar to that observed in wild-type receptors (n = 5, P = 0.18, unpaired Student’s t-test) (Fig. 4d). Taken together, these tests support the hypothesis that H^+^-dependent and Zn^2+^-dependent potentiation of GluN1/GluN3A currents act through separate residues. Thus, the H^+^-dependent potentiation we report here reveals a novel allosteric site, most likely located in the LBD layer.

### Residues facing the LBD heterodimer interface mediate proton-dependent potentiation

**Figure 5:**
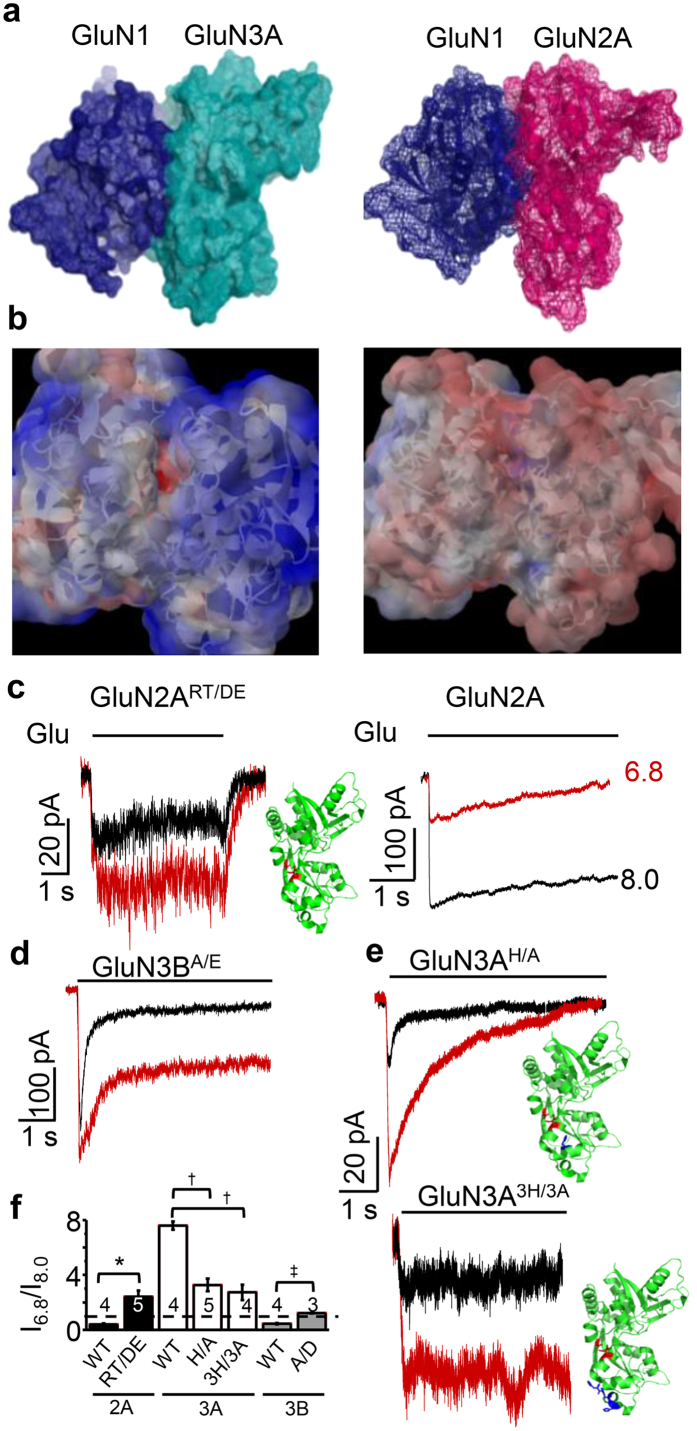
Charged residues in the ligand-binding domain can transfer proton potentiation. (**a**) Atomic models and (**b**) calculated electrostatic potentials for LBD heterodimers of GluN1/GluN3A (left) and the GluN1/GluN2A (PDB: 2A5T) (right); surface charges are highlighted in red (negative) and blue (positive). (**c**–**e**) Whole-cell current traces recorded at pH 8.0 (black) or pH 6.8 (red) from the indicated proteins; cartoons of the LBD (green) highlight the putative proton sensor (red) and the neighbouring His residues (blue); (**f**) Summary of results; values are means ± SEM; sample size is in/above bars; *P < 0.05 relative to GluN2A (2A, WT), ^†,^P < 0.05 relative to GluN3A (3A, WT), and ^‡^P < 0.05 relative to GluN3B (3B, WT).

Residues located at the dimer interface within the LBD play critical roles in the kinetic properties of all iGluRs investigated so far. Eminently, in AMPA and kainate receptors^23^,^24^,^38^,^39^,^40^, but not in canonical NMDA receptors^25^, perturbations in this region with ligands or mutations alter the macroscopic current desensitisation. In particular, for GluK3 kainate receptors, Zn^2+^ binding at this interface slows current desensitisation and accelerates current recovery^26^. Based on this knowledge, we investigated the LBD dimer interface as a potential locus for proton-dependent modulation. To survey residues within the LBD that may experience protonation/deprotonation equilibria in the physiologic range of pH values, we produced a model for the GluN1/GluN3A LBD heterodimer based on published crystallographic structures of the GluN1/GluN2A LBD heterodimer (PDB: 2A5T)^41^ and the GluN3A LBD monomer (PDB: 2RC7)^42^ (Fig. 5a). Electrostatic surface potentials calculated using the Adaptive Poisson-Boltzmann Solver^43^ revealed a cluster of anionic surface charges at the dimer interface of GluN1/GluN3A but not of GluN1/GluN2A (Fig. 5b). In this region of GluN1/GluN3A, we identified unbalanced anionic charges at E781 of GluN1, and at E871 and D804 of GluN3A. In contrast, in GluN1/GluN2A, this region was electrically neutral because the corresponding residues in GluN2A are R692 and T759, and the charges of GluN1 E781 and GluN2A R692 form a neutral salt bridge across subunits.

To test whether these anionic residues are part of the pH sensor, we exchanged the homologous residues in GluN3A with those present in GluN2A to produce GluN3A^E871T^ and GluN3A^D804R^, reasoning that the swap will abolish the proton sensitivity of GluN1/GluN3A currents. However, we were unable to record currents from cells co-transfected with GluN1 and GluN3A^E871T^, GluN3A^D804R^, or GluN3A^E871T/D804R^ (data not shown). Moreover, simply neutralizing the anionic charge of either or both these two residues with isosteric substitutions (GluN3A^E871Q^, GluN3A^D804N^, or GluN3A^E871Q/D804N^) also resulted in complete loss of glycinergic currents (data not shown). We surmise that by controlling electrostatic interactions across subunits, the negative charges of GluN3A E871 and D804 may be required for critical receptor functions such as glycine-dependent gating or/and oligomerization. To overcome this obstacle, we took the reverse approach and introduced in GluN2A the residues present at these positions in GluN3A to produce GluN2A^R692D/T759E^. Remarkably, acidification from pH 8.0 to 6.8 potentiated GluN1-1b/GluN2A^R692D/T759E^ currents ~2.5-fold (n = 5, P = 0.008, paired Students t-test) (Fig. 5c left, and 5f). This pH effect is the opposite of that observed for wild-type GluN1-1b/GluN2A receptors, whose currents are *reduced* ~2-fold for a similar pH change^28^ (n = 4, P = 0.005, unpaired Student’s t-test) (Fig. 5c right, and 5f). Therefore, our new result shows that introducing negative charges at R692 and T759 impart novel proton sensitivity onto GluN2A-containing receptors. Although these results support the hypothesis that, in GluN3A, E871 and D804 mediate proton-dependent potentiation, GluN1-1b/GluN2A^R692D/T759E^ produced very small currents, consistent with previous reports that in GluN1/GluN2A perturbations at the LBD dimer interface strongly impair gating^25^. Therefore, we sought additional evidence for a possible role of GluN3A E871 and D804 in mediating proton-dependent potentiation.

Like GluN1/GluN2A, but in contrast to GluN1/GluN3A, GluN1/GluN3B currents are inhibited by protons^32^ (Fig. 1b). We reasoned that if these receptors tolerate residue swapping without substantial impact on gating, they may provide the needed information. To identify the residues in GluN3B corresponding with the hypothesised GluN3A proton sensor, we compared the primary sequences of these subunits in their lower LBD regions and found substantial identity (80%). Specifically, of the two negatively charged residues hypothesised to be part of the proton sensor of GluN3A, only one differed, such that it was neutral (GluN3B A704) rather than anionic (GluN3A D804). We therefore tested the effect of pH on GluN3A- and GluN3B-containing receptors with swapped residues at this position.

Similar to the other substitutions tested at this location (D804R and D804N, see above), GluN3A^D804A^ resulted in complete loss of function. In contrast, we were able to record glycinergic currents from GluN1/GluN3B^A704D^ (n = 3) and found that in contrast to their wild-type counterparts (n = 4), which were 2-fold inhibited when pH changed from 8 to 6.8 (Fig. 1b), the peak current response of these currents increased 1.21 ± 0.03-fold (P = 3 × 10^−5^, unpaired Student’s t-test) (Fig. 5d,f). Therefore, introducing a single negative charge at the LBD dimer interface of GluN1/GluN3B receptors reversed the effect of protons on these receptors from inhibition (Fig. 1b) to potentiation (Fig. 5d,f). These results represent additional evidence that negatively charged residues located at the LBD heterodimer interface may mediate the proton-dependent potentiation of GluN1/GluN3A currents.

Given that GluN1/GluN3A currents were strongly sensitive to small deviations from physiologic pH, we reasoned that proximal histidine residues, whose protonation equilibria occur in this range as well, might also affect the receptors’ sensitivity to external pH. Using the LBD heterodimer model (Fig. 5a,b), we identified visually four histidine residues in GluN3A facing the dimer interface: one isolated (H814), and three clustered (H781, H786, and H787). The single-residue mutation H814A reduced substantially proton potentiation (I_6.8_/I_8.0_) from ~7-fold for wild-type currents (n = 4) to ~3-fold for the H/A mutant (n = 5, P = 0.014, unpaired Student’s t-test), and the triple-substitution, H781A/H786A/H787A, reduced potentiation to ~2.5-fold (n = 4, P = 0.014, unpaired Student’s t-test) (Fig. 5e,f).

In summary, our structure-based mutagenesis results indicate that: 1) histidine residues facing the LBD heterodimer boundary of GluN1/GluN3A receptors contribute substantially to the degree of proton-dependent potentiation, and that 2) anionic residues specific to GluN1/GluN3A can reverse the effects of protons from inhibition to potentiation when substituted into GluN2A- or GluN3B-containing receptors. Based on these results, we postulate that protons most likely exert their potentiation effects by modifying residues that face the LBD heterodimer interface which likely stabilize this dimer, similar to GluK3 homomeric receptors^26^.

### Effect of GluN1/GluN3A currents on the cellular resting membrane potential

Given that GluN1/GluN3A receptors have high glycine affinity and glycine is ubiquitous in brain interstitia, the prevailing view is that GluN1/GluN3A receptors are tonically active *in vivo*^42^, and physiologic or pathologic conditions that alter these basal levels may also alter the cellular resting membrane potential. In brain, extracellular glycine concentrations fluctuate between values as low as 5–15 μM during rest and can reach up to 1 mM, during periods of high synaptic transmission^44^,^45^. To determine whether, and if so, how, acidic pH transients affect the tonic level of GluN1/GluN3A currents, we recorded whole-cell currents from HEK293 cells expressing GluN1/GluN3A receptors during prolonged applications (>30 s) of low (10 μM) or high (500 μM) glycine concentrations at pH 7.4 and pH 6.8. To avoid possible contributions from proton-gated ASIC channels, which are endogenous to HEK cells (EC_50_, pH 6.45)^46^, in these experiments extracellular solutions also contained amiloride, an ASIC inhibitor (20 μM, IC_50_ = 2.2 μM)^46^.

**Figure 6:**
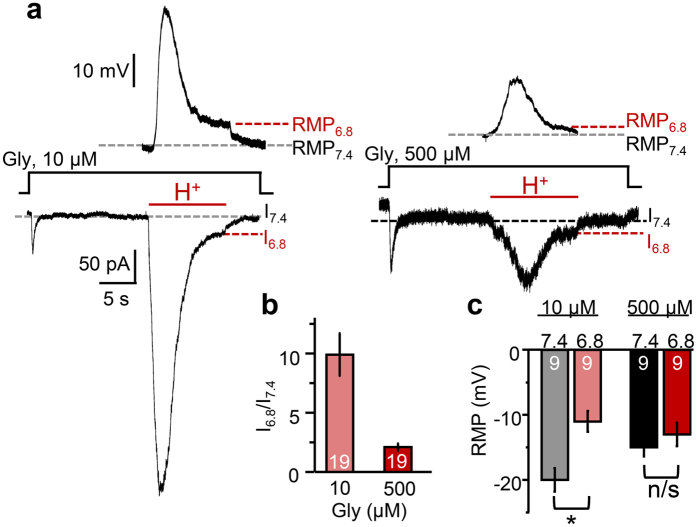
Protons resensitise tonic glycinergic currents and can depolarize the cellular membrane. (**a**) Whole-cell currents and membrane potentials were recorded in high (500 μM) or low (10 μM) ambient glycine concentrations from HEK 293 cells expressing recombinant GluN1/GluN3A receptors at pH 7.4 and during transient acidification (pH 6.8, red bar). (**b**) Summary of proton-dependent changes in equilibrium current (I_6.8_/I_7.4_) at two glycine concentrations; (**c**) summary of measured resting membrane potentials; values are means ± SEM; sample size is in bars; *P < 0.05; n/s, P > 0.05.

In physiologic pH (7.4), glycine application elicited small currents, which desensitised to almost undetectable steady-state values (I_7.4_), as in our previous experiments. In cells expressing GluN1/GluN3A receptors, but not in untransfected cells (Supplementary Fig. 1), shifting the pH to 6.8 (10 s) produced an initial increase in the glycinergic current, which equilibrated slowly to a new steady-state level (I_6.8_) (Fig. 6a). Relative to the tonic level measured in physiologic pH (I_7.4_, before or after the pH challenge), the tonic current measured during the acidic pulse (I_6.8_) was ~10-fold higher in low glycine (n = 19, P = 2 × 10^−6^, paired Student’s t-test), and only ~2-fold higher in high glycine (n = 19, P = 0.004, paired Student’s t-test) (Fig. 6b and Table 2). These results show that in cells expressing GluN1/GluN3A receptors, acidic shifts can potentiate tonic glycinergic currents and the magnitude of this potentiation is dependent on the background glycine concentration, such that the change is much larger in low than in high glycine concentrations.

We next examined whether these changes in basal glycinergic currents may reflect in measurable fluctuations in whole-cell membrane potential. Using identical stimulation protocols in low background glycine concentration, we found that relative to the resting membrane potentials measured in physiologic pH (RMP_7.4_), those measured under acidic pH (RMP_6.8_) were significantly depolarized: RMP_7.4_, −20 ± 1.8 mV versus RMP_6.8,_ −11 ± 1.6 mV (n = 9, P = 9 × 10^−6^, paired Student’s t-test) (Fig. 6a,c and Table 2). However, similar measurements in high background glycine concentration revealed no significant pH-dependent change: RMP_7.4_, −15 ± 1.4 mV versus RMP_6.8,_ −13 ± 1.8 mV (n = 9, P = 0.07, paired Student’s t-test) (Fig. 6a,c and Table 2). These results show that acidic shifts depolarize GluN1/GluN3A-expressing cells in a glycine concentration-dependent manner.

## Discussion

We report the novel observation that glycinergic GluN1/GluN3A currents are robustly potentiated by external acidification within the range of pH fluctuations reported to occur in brain interstitia^14^,^47^. This effect is unique within the NMDA receptor family, whose all other members produce currents that are inhibited by acidic shifts^19^,^28^,^32^. Our results indicate that protons potentiate GluN1/GluN3A currents largely by reducing their desensitisation, and that specific protonatable residues facing the LBD dimer interface can transfer H^+^-dependent potentiation onto GluN1/GluN2A and GluN1/GluN3B receptors. Last, we found that in low background levels of glycine, protons can depolarize GluN1/GluN3A-expressing cells. These observations provide new insights into the possible function and biologic roles of glycinergic GluN1/GluN3A currents and provide a new and powerful pharmacologic tool to reveal these currents in native preparations.

Protons potentiate GluN1/GluN3A currents through a novel allosteric site. Protons strongly altered the current amplitude and kinetics (Fig. 2) but did not alter the current/voltage relationship (Fig. 4c), thus indicating that protons modified gating rather than permeation properties. Specifically, we found that the 3-fold increase in peak current produced by shifting the pH from 7.4 to 6.8, slowed the desensitisation time constant 3-fold, and accelerated the recovery time constant 4-fold, an indication that the observed increase in current was largely due to decreased desensitisation. Further, we found that the site mediating this effect is distinct from all other known NMDA receptor allosteric modulators^9^.

Several physiologic (H^+^, Zn^2+^, polyamines) and pharmacologic (ifenprodil) modulators bind in the NTD of GluN1/GluN2 receptors and strongly alter the magnitude and kinetics of macroscopic currents^7^. In particular, the sensitivity of GluN1/GluN2 receptors to proton inhibition is strongly affected by regulated expression of GluN1-a or -b splice variants, which differ in their NTD sequences^28^. In contrast, we found that all naturally occurring GluN1/GluN3A variants, whether differing in their NTD or CTD sequences, were equally sensitive to H^+^-dependent potentiation (Fig. 3). Together these results indicate that GluN1/GluN3A proton sensitivity is independent of NTD and CTD residues, and therefore is most likely mediated by residues in the LBD.

Zn^2+^ and glycine are two known GluN1/GluN3A modulators that bind LBD residues^9^,^42^. Four lines of evidence indicate that the proton-site is distinct and thus novel. First, our results show that external protons shifted the glycine dose response only modestly (Fig. 4a). Second, a single residue substitution in the agonist-binding cleft of the GluN1 (F484A), which abolishes Zn^2+^-dependent potentiation of GluN1/GluN3A currents, had no effect on H^+^-dependent potentiation (Fig. 4d). Third, unlike Zn^2+^, which relieves voltage-dependent block by Ca^2+^ (ref. 9), acidification had no effect on the current/voltage relationship (Fig. 4c). Last, at H^+^ concentrations that produced maximal potentiation, Zn^2+^ remained effective and further increased the response. Based on these results we conclude that protons potentiate GluN1/GluN3A currents through a novel allosteric site. Results from structure-based mutagenesis suggest that this site may reside at the LBD heterodimer interface.

The atomic architecture of GluN1/GluN3A receptors is presently unknown. We generated a homology model for the GluN1/GluN3A LBD heterodimer based on the structures published for the GluN1/GluN2A LBD heterodimer and for the isolated GluN3A LBD^41^,^48^. In contrast to the GluN1/GluN2A LBD structure, the GluN1/GluN3A LBD model revealed a local density of negative surface charge across the solvent-accessible heterodimer boundary; in addition, several histidine residues pointed towards this boundary (Fig. 5b). We hypothesised that protonation of these residues, by reducing repulsions between subunits, may increase the dimer stability as a mechanism for their potentiating effect, similar to GluK3 homomers^26^. Substituting the anionic residues in GluN3A (E871 and/or D804) resulted in complete loss of GluN1/GluN3A function; therefore, we were not able to test their role in proton sensing with this approach. However, substituting GluN3A histidine residues (H781, H786, H787, and H814) produced functional receptors, which were significantly less sensitive to H^+^-dependent potentiation. Given that the histidine mutants also had altered gating, it is unclear whether the decreased magnitude of the H^+^-dependent potentiation reflected a change in proton binding ability at these sites or a role of the histidine side chains in receptor gating. However, results clearly show that residues elsewhere continued to confer H^+^-dependent potentiation onto the histidine mutants.

Therefore, we focused on GluN3A E871 and D804 as putative proton sensors and took the reverse approach of introducing these negative charged residues at homologous locations in GluN1/GluN2A and GluN1/GluN3B, for which protons are normally inhibitory^19^,^28^,^32^. Remarkably, protons potentiated currents from these ‘charged’ receptors, suggesting that proton potentiation through this novel allosteric site overpowered the effects of proton binding at the native inhibitory site(s). Together, our results from structure-based mutagenesis suggest that exposed residues at the LBD heterodimer boundary are critical to GluN1/GluN3A receptor function and that protonatable residues at this location may represent a novel allosteric site that mediates H^+^-dependent potentiation of GluN1/GluN3A currents.

The gating mechanism of GluN1/GluN3A receptors is presently unknown. In AMPA and kainate receptors, the LBD dimer interface is a major modulator of channel activity and a prominent molecular determinant of current desensitisation^7^. Specifically, diffusible ligands that bind at this interface promote LBD dimer formation in solution and increase receptor activity by reducing their desensitisation. In contrast, the LBD heterodimer interface of GluN1/GluN2 receptors is larger and more stable^41^,^49^,^50^; and further stabilizing this interface with engineered disulphide bridges prevents receptor activation with no effect on desensitisation kinetics^25^. In this context, our finding that H^+^-dependent potentiation, which relies most likely on residues located at the dimer interface, correlated with marked decrease in GluN1/GluN3A current desensitisation and may indicate that the activation mechanism of these receptors resembles that of non-NMDA receptors. This hypothesis is also consistent with the observation that like AMPA and kainate receptors, but not GluN1/GluN2 NMDA receptors, GluN1-1a/GluN3A receptors desensitise deeply, with only a small residual current (~10% of peak) at equilibrium^29^.

However, in the absence of a kinetic model it is premature to speculate if the observed changes in macroscopic current desensitisation reflect changes in the stability of microscopic desensitised states. For example, GluN1-4a^F/A^/GluN3A and GluN1/GluN2A^RT/DE^, which produced non-desensitising currents, remained sensitive to H^+^-dependent potentiation. Therefore, to establish the mechanism by which protons potentiate GluN1/GluN3A currents it will be necessary to know more about the sequence and rates of conformational changes that constitute these receptors’ gating reaction. This information will help to further understand how glycine and proton transients acting on GluN1/GluN3A receptors control cellular physiology.

The physiologic role of glycinergic NMDA receptors is a matter of speculation and some debate, mostly because reports of native glycinergic excitatory currents remain scarce^8^,^51^,^52^. Because GluN1/GluN3 receptors produce deeply desensitising currents^29^ and glycine is omnipresent in brain interstitia^44^, a viable hypothesis is that GluN1/GluN3 receptors modulate the resting membrane potential depending on the ambient level of glycine. Our results support this hypothesis. We found that in physiologic pH (7.4) and low glycine concentration (10 μM) increasing glycine concentration to 500 μM slightly depolarized HEK cells expressing GluN1/GluN3A receptors (Table 2). In addition, we discovered that increasing the proton concentration to pH 6.8 was more effective at depolarizing these cells than increasing glycine. Based on these results we suggest that GluN1/GluN3A receptors may serve to modulate cellular membrane potentials in response to fluctuations in glycine or proton concentration.

Extracellular glycine and proton concentrations vary in brain during physiologic events and abnormal values accompany certain pathologies^14^,^17^,^44^,^45^,^47^,^53^,^54^,^55^. Notably, acidification of brain interstitia occurs in stroke and schizophrenia^16^,^17^,^18^; likewise, glycine supplementation alleviates negative symptoms associated with schizophrenia^56^,^57^,^58^,^59^. It is unclear where GluN1/GluN3A receptors are expressed and how their expression is regulated, however, changes in GluN3A expression occur in ischemia, schizophrenia, Huntington’s disease, and addiction^10^,^11^,^12^,^13^. Also unclear is whether ectopic expression of GluN3A aggravates the pathology or represents a protective response; regardless, the robust potentiation of GluN1/GluN3A currents we describe here may provide a direct link between the two and represents a springboard into deciphering how these are related. Further, the proton sensor we identify here may represent both an investigative tool and a therapeutic target in diseases where brain pH and/or expression of GluN3A are disrupted.

GluN3 subunits are the most recent additions to the iGluR family; they have been assigned to the NMDA receptor class based on their high sequence homology with GluN1 subunits, and based on their ability to decrease the magnitude of glutamate-gated currents from cells expressing GluN1/GluN2 iGluRs, but not others^4^,^6^. These observations drove the initial hypothesis that where expressed, GluN3 subunits co-assemble with GluN1 and GluN2 subunits to form triheteromers with biophysical properties distinct from those of GluN1/GluN2 diheteromers, i.e. reduced current amplitude, reduced Ca^2+^ permeability, and reduced voltage-dependent Mg^2+^-block. Later, it was discovered that GluN3 subunits have ligand-binding properties similar to the GluN1 rather than the GluN2 subunits, i.e. they bind glycine but not glutamate, NMDA, or AP5^48^. This observation provided an explanation for the sensitivity of recombinant GluN1/GluN3 channels to glycine and insensitivity to glutamate-site agonists and antagonists; further, glycinergic currents were also insensitive to NMDA receptor class-specific blockers (Mg^2+^, memantine, and MK-801)^8^. These important findings provided the first functional tools to examine whether glycinergic excitatory receptors are expressed in native tissues. Recently, these functional criteria helped to demonstrate a role for glycinergic GluN1/GluN3B receptors in hippocampal metaplasticity^60^.

Previous reports and our results show that in physiologic pH, glycine elicits small currents that desensitised quickly (τ_D_, ~50 ms) and deeply (I_ss_/I_pk_, ~10%); therefore, it is plausible that contamination with low levels of glycine, which is prevalent in native preparations, prevents the detection of GluN1/GluN3A glycine-activated currents. Our observation that protons strongly and uniquely potentiate glycinergic GluN1/GluN3A currents represents a powerful pharmacologic tool to probe their occurrence in native preparations and their contributions to cellular physiology. We anticipate that this new knowledge will facilitate the detection of native GluN1/GluN3A-mediated currents and will accelerate the characterisation, in a physiological context, of this still enigmatic receptor subtype.

## Experimental Procedures

### Molecular biology

Plasmids encoding the following rat NMDA receptor subunits were gifts: GluN1-1a (NR1-1a, U08261) from R. Wenthold (US National Institutes of Health, Bethesda, MD), GluN1-1b (NR1-1b, U08263) from S. Traynelis (Emory University, Atlanta, GA), GluN1-2a (NR1-2a, U08262) and GluN1-3a (NR1-3a, U08265) from J. Woodward (Medical University of South Carolina, Charleston, SC), GluN1-4a (NR1-4a, U08267) from R. S. Zukin (Albert Einstein College of Medicine, Bronx, NY), and GluN3A (NR3A, NM_001198583) and GluN3B (NR3B, NM130455) from S. Lipton (University of California, San Diego, San Diego, CA). Each coding sequence was sub-cloned into pcDNA3.1(+), except GluN1-4a, which was sub-cloned into pRK5. Plasmids were isolated and purified (QIAGEN, Valencia, CA). GluN1 C-terminal truncations (GluN1^838stop^)^34^, GluN1 and GluN3A N-terminal deletions, and all point mutations were produced using site-directed mutagenesis (QuikChange II XL site-directed mutagenesis kit, Agilent Technologies, Santa Clara, CA). Residue notations refer to the full cDNA-encoded sequence, which includes the signal peptide. For the GluN1 and GluN3A N-terminal domain truncations (GluN1^ΔNTD^ and GluN3A^ΔNTD^), the signal peptide was retained to facilitate efficient trafficking (GluN1 M1 through D23; GluN3A M1 through Q39); in GluN1^ΔNTD^, P24 through D376 were deleted; in GluN3A^ΔNTD^, I40 through S493 were deleted. Both deletion protocols used a loop-out procedure. For the GluN3A C-terminal truncation, a stop codon was introduced at H956, with the resulting sequence M1 through V955. All coding sequences were verified by full-insert sequencing.

### Recombinant protein expression

Human embryonic kidney (HEK) 293 cells (ATCC CRL-1573), a gift from A. Auerbach (University at Buffalo), were maintained in Dulbecco’s Modified Eagle’s Medium (DMEM) (Invitrogen, Grand Island, NY) supplemented with 10% fetal bovine serum and 1% penicillin-streptomycin; were maintained in 5% CO_2_ at 37 °C; and used for transfections between passages 20–35. Calcium phosphate-mediated transfections^61^ were done by incubating cells (40–50% confluence) for 3 hours with transfection mixtures. These contained (in mM): 140 NaCl, 5 KCl, 0.75 Na_2_HPO_4_, 6 sucrose, 125 CaCl_2_, 25 HEPES/NaOH, pH 7.05, and plasmids expressing NMDA receptor subunits as indicated: GluN1 (1 μg) and either of GluN3A (2 μg), GluN3B (2 μg), or GluN2A (1 μg), and GFP (1 μg) per four 35 mm-dishes. Following incubation, cells were washed with excess PBS, and incubated in 2 mL DMEM supplemented with 2 mM MgCl_2_ or 100 μM D-serine to prevent excitotoxicity. Transfected cells were used 24–48 hours thereafter for electrophysiological measurements.

### Electrophysiology

Whole-cell currents were recorded from HEK 293 cells with the patch-clamp method using fire-polished pipettes (5–10 MΩ) filled with intracellular solution containing (in mM): 135 CsCl, 33 CsOH, 2 MgCl_2_, 1 CaCl_2_, 10 HEPES, 11 EGTA at pH 7.4 (CsOH). Cells were perfused with extracellular solution containing (in mM): 150 NaCl, 2.5 KCl, 0.5 CaCl_2_, and 10 HEPBS (pH 8.0), HEPES (pH 7.4–7.0), or PIPES (pH 6.8–6.7) with or without 0.5 glycine. For experiments with external zinc, the extracellular solutions contained (in mM): 150 NaCl, 2.5 KCl, 0.5 CaCl_2_, 10 tricine, 10 HEPES or PIPES, and ZnCl_2_ to produce the free-Zn^2+^ concentrations indicated. Tricine-buffered free-Zn^2+^ concentrations were calculated using MaxChelator (C. Patton, Stanford University, personal communication) and took into account ionic strength of the solution, temperature, pH, and tricine concentration. For proton-activated currents, 20 μM amiloride was included in the external recordings solutions to exclude the activation of acid-sensing ion channels present in HEK 293 cells^46^.

To prevent non-specific receptor activation by contaminating levels of glycine^62^,^63^, double-distilled deionized ultrapure water (Fisher Scientific, Hampton, NH) was used when preparing all solutions. Prior to use, all glassware and related equipment were soaked in this water >24 hours to leech contaminating glycine. Perfusate flow was controlled through a pressure-driven pinch valve perfusion system (BPS-8, ALA Scientific Instruments Inc., Westbury, NY) and solution exchange and voltage protocols were generated in Clampex (Molecular Devices, Sunnyvale, CA). Zinc experiments were done in solutions maintained at 25 °C under stringent temperature control using a heated and cooled perfusion system (HCPC, ALA Scientific Instruments Inc., Westbury, NY), as necessary for the accurate control of free-Zn^2+^ concentrations in tricine buffer.

Inward current was recorded while clamping cells at −70 mV, sampled at 5 kHz, and low-pass filtered at 2 kHz (Digidata 1440A, Molecular Devices, Sunnyvale, CA). Typically, 3–10 traces were recorded from each cell, averaged, and analysed in Clampfit (Molecular Devices, Sunnyvale, CA). The weighted time constant of macroscopic desensitisation (τ_D_) was calculated by fitting the recorded trace between maximum (I_pk_) and steady-state (I_ss_) current amplitudes with a declining biexponential function. The extent of desensitisation was calculated as the ratio of I_ss_ to I_pk_. Dose response profiles were constructed from I_pk_ values recorded in several proton concentrations and were quantified by fitting the Hill equation to these data.

### Molecular Modelling

Molecular modelling for the GluN1/GluN3A heteromeric ligand binding domain core was done with SYBYL X software (Certara USA, Princeton, NJ) run on computers with sufficient processing power and dual Windows 7 and Mac operating systems. The GluN1/GluN2A structure (PDB: 4A5T)^41^ was used as a scaffold upon which the GluN3A ligand binding domain structure (PDB: 2RC7)^42^ was aligned (to GluN2A), resulting in a preliminary model for the GluN1/GluN3A heteromeric ligand binding domain core. This preliminary structure was subject to iterative energy minimization using AMBER FF99 until the structure converged to an acceptable energy minimum. For the final minimized structure and for the GluN1/GluN2A crystal structure, we calculated the solvent accessible surface potentials using the Adaptive Poisson Boltzmann Solver (APBS) software^43^. We used this model to guide mutagenesis experiments. Images were exported from APBS as surface potential images or were prepared in PyMol (www.pymol.org).

## Additional Information

**How to cite this article**: Cummings, K. A. and Popescu, G. K. Protons Potentiate GluN1/GluN3A Currents by Attenuating Their Desensitisation. *Sci. Rep.* **6**, 23344; doi: 10.1038/srep23344 (2016).

## Supplementary Material

Supplementary Information

## Figures and Tables

**Table 1:** pH dependence of glycinergic whole-cell current desensitisation kinetics.

**pH**	**Desensitisation**	**Recovery**	
	**τ**_**D**_**(ms)**	**τ**_**rec**_**(s)**	**t**_**1/2**_**(s)**
6.8	133 ± 26*	2.1 ± 0.2*	4.6 ± 0.2*
7.4	52 ± 13	8.0 ± 0.2	8.4 ± 0.3

*P < 0.05 relative to pH 7.4 (paired Student’s t-test).

**Table 2:** pH and glycine dependence of whole-cell current and potential.

**[glycine] (μM)**	**n**	**I**_**6.8**_**/I**_**7.4**_	**RMP**_**7.4**_ **(mV)**	**RMP**_**6.8**_ **(mV)**
10	9	9.9 ± 1.8	−20 ± 1.8	−11 ± 1.6*
500	9	2.1 ± 0.3	−15 ± 1.4^#^	−13 ± 1.8

*P < 0.05 relative to 7.4; ^#^P < 0.05 relative to 10 μM glycine; (paired Student’s t-test).
