# Supplementary material for: Protons Potentiate GluN1/GluN3A Currents by Attenuating Their Desensitisation
Source: Sci Rep. 2016 Mar 22;6:23344. doi: 10.1038/srep23344 (PMC4802338; doi:10.1038/srep23344)
Supplement: Supplementary Information [file srep23344-s1.pdf]

Protons Potentiate GluN1/GluN3A Currents by Attenuating Their Desensitisation

Kirstie A Cummings and Gabriela K Popescu*

Department of Biochemistry, University at Buffalo, SUNY, Buffalo, NY 14214, USA

*Corresponding author: Gabriela K Popescu, popescu@buffalo.edu

Supplementary Figure S1

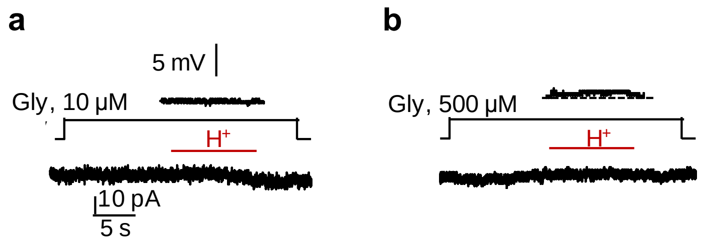

Figure S1. Current and membrane potential recordings from untransfected HEK 293 cells. Whole-cell currents and membrane potentials were recorded in (**a**) low (10 µM) or (**b**) high (500 µM) ambient glycine concentrations from untransfected HEK 293 cells at pH 7.4 and during transient acidification (pH 6.8, red bar).
